# What does good look like? Officers’ perception of the ideal law enforcer

**DOI:** 10.3389/fsoc.2025.1568487

**Published:** 2025-04-24

**Authors:** Stephen W. Bell, Jennifer L. Cliff

**Affiliations:** ^1^Criminal Justice Program, School of Professional Studies, University of Kansas, Lawrence, KS, United States; ^2^Psychology Department, Mount Marty University, Yankton, SD, United States

**Keywords:** police, law enforcement, humanity, moral courage, resilience, guardian, warrior

## Abstract

The purpose of this basic qualitative study was to explore how law enforcement officers describe the attributes, characteristics, and qualities that combine to form the archetype of the ideal law enforcer. Convenience sampling was used to recruit 22 law enforcement officers. Data were collected through semi-structured interviews and thematically analyzed. While the participating officers were candid about the traits they perceived to be ideal among law enforcers, interestingly, these officers tended to avoid topics of physicality. The bulk of the coded data revealed themes that related to personality characteristics. Findings revealed that law enforcement officers categorized their self-described ideal traits of other officers into three primary themes: (1) Humanity and Emotional Intelligence, (2) Moral Courage, and (3) Resilience and Occupational Effectiveness.

## Introduction

As a publicly funded occupation, law enforcement carries certain expectations of excellence. Members of the taxpaying public demand a certain level of vigilance, fairness, trustworthiness, and legitimacy (Charman et al., 2022; Perry and Jonathan-Zamir, 2020). The roles assumed in policing are often discretionary and somewhat unsupervised, as the decisions made by officers come with immediate autonomy and are scrutinized afterward (Bell and Adams, 2023; Huff, 2021). Additionally, there is an expectation that law enforcement officers take on different roles, such as counselor, enforcer, and problem solver (Zakimi et al., 2022). These expectations are not limited to the exterior perspectives of the community. Peer groups also develop internal demands. Regardless of the perspective or the occupation, a struggle exists to define a hypothetical ideal model of the subject. Among law enforcement officers, the qualities of the ideal police officer are not always apparent, nor is there a consensus regarding the existence of a so-called police personality (Ten Eyck, 2024). The ambiguity is exacerbated further by how the different promotional and rank-based roles experienced within a career can alter that person’s perspectives. Understanding that opinions and perspectives may not remain constant as roles, responsibilities, and rank change is crucial.

The researchers in the current study aimed to examine these ideal characteristics of law enforcement officers from the unique perspective of their fellow officers. A total of 22 officers were interviewed to provide data regarding their perceptions of a so-called good officer. The current study gathered data through the perspective of law enforcement officers in the Western United States, a region that has recently experienced a thriving defund the police movement and calls for police reforms in de-escalation and transparency (Cobbina-Dungy and Jones-Brown, 2023; Cook and Fortunato, 2023; Fine et al., 2022; Plummer et al., 2024). This movement would seemingly conflict with the normative values of police officers and impact the perceptions of what so-called *good* looks like among officers and the community. The unit of analysis within the study consisted of the individual officers’ perceptions, which were shaped by their lived experiences with community expectations and organizational norms.

This was the genesis of the study’s title, which was borrowed from the academic discipline of education. Educators understand that multiple people may look at the same assignment and grade it with different criteria. To avoid misunderstandings and ambiguity in feedback, academia has adopted rubrics (Chowdhury, 2019; Krebs et al., 2022). A common phraseology associated with the use of rubrics is “*What does good look like?”* (Hooper et al., 2024; Landoni et al., 2022). This phenomenon can be transferred to the concept of discovering what characteristics law enforcement officers attribute to the ideal police officer, with the understanding that there are multiple occupational demographics within one career. There is more than one police officer career point. There are academy recruits, trainees, field training officers, supervisors, managers, and administrators (Townsend and Loudoun, 2024). For the purposes of this study, we focus on two of these points: officers and supervisors.

Modern law enforcement officers are required to assume a variety of occupational roles within their daily duties. These roles do not always comport; in fact, at times, they may conflict in the way they are expected to wear the proverbial hats of enforcer, helper, mediator, and mental health worker (Bailey et al., 2022; Zakimi et al., 2022). Given these various functions, the researchers in the current study viewed the data through the lens of a multiple-role heuristic. The participants’ perceptions of the ideal law enforcer are guided by their awareness of differing occupational responsibilities. By viewing the traits that fellow officers identify as so-called *ideal*, researchers and administrators can more effectively understand the most desired traits from an internal perspective.

The perspective of fellow officers provides a unique lens through which to view the perceived ideal traits of a law enforcer. Understanding internal biases and perspectives is especially significant when compared to public perceptions and organizational policies. As agencies establish expectations in hiring and training, identifying how fellow officers describe the ideal officer provides an opportunity for administrators to comparatively analyze these descriptions with the organizational expectations they hold. By comparing the organizational expectations, the desired norms of the administration emerge but may not necessarily support the established norms embodied by the officers within the agency. Ensuring the alignment of the desired norms and actual norms is crucial to organizational cohesion.

## Literature review

Previous research into the ideal characteristics of law enforcement officers has a distinct trajectory. There are two primary strands: the way officers choose to portray themselves and the way the public perceives them. As the authors of the current study examined the previous literature, they found that the ways in which officers choose to portray themselves through their actions and enforcement decisions fell into two distinct categories. These two categories are most commonly described as warriors and guardians (Clifton et al., 2021; Inzunza and Wikström, 2020; McLean et al., 2020; Murphy and McCarthy, 2024). Strah et al. (2023) defines the guardian mindset by using the image of communication, not commands. The guardians see their occupational priority as a duty to protect through a focus on crisis intervention, de-escalation, and procedural justice (Simon, 2023; Strah et al., 2023). Conversely, the warrior mindset is embodied by officers who see police work as a battle between good and evil. These officers may fail to consider their role as community representatives (McLean et al., 2020).

Inzunza and Wikström (2020) found that these two mindsets of warrior and guardian may be a product of nurture versus nature. They examined new police recruits and found a correlation between youth and inexperience to the desire for positive relationships with the community. Not all policing traits were defined by the level of experience. For example, the ability to differentiate between truthful and untruthful statements was examined by Manzanero et al. (2015), and they found no difference between experienced and inexperienced officers.

Regarding officers who displayed characteristics aligned with a warrior mindset, McCarthy et al. (2024) found that these officers were more likely to see danger in ambiguous situations and support the use of physical force to quell the perceived threats. In contrast, guardian-centered officers were likelier to show restraint in the face of perceived danger. Clifton et al. (2021) supported these findings. They reported that instilling the guardian mindset in younger officers could lead to a sense of genuine trust between communities and their law enforcement officers.

In current literature, Murphy and McCarthy (2024) supported previous research and found two orientations of law enforcement officers that they described as conceptually distinct. They used the guardian label to describe officers who embraced procedural justice and the warrior label for officers who embraced coercive policing techniques. Oliveira and Jackson (2021) studied the topic of coercive policing and found that it leads to alienation and public detachment.

The next strand of literature that the authors examined was the ways officers are perceived by others. This type of categorization differed from the previous strand based on the so-called eye of the beholder. Rather than a conscious choice to behave in a particular manner, this strand focuses on how their peers and the public judge officers regardless of the persona they attempt to portray.

Sjöberg et al. (2024) conducted research specifically focused on officers who were assigned to Special Weapons and Tactics (SWAT) teams, which have unusually high contact with violent offenders. After surveying 159 male SWAT officers, they found that a high level of discipline and a low level of vulnerability were preferred. Menton et al. (2024) also studied specific officer types when examining the characteristics, including a lack of integrity, that correlate with negative field performance among recruit officers. Azzahrah et al. (2024) researched ethics as mandated by codified documents among law enforcement agencies. They found that professionalism, defined as knowledge and technical ability, were highly desired traits from an organizational perspective.

When continuing the review of the literature regarding the ideal characteristics of law enforcers, Schuck and Rabe-Hemp (2024) surveyed community members to examine how traditionally feminine traits would be viewed if they were elevated in policing. They found that compassion was a highly desired trait for officers. In related research, Sunde (2024) examined how officers perceive de-escalation and its benefits. The findings showed a relationship between de-escalation and compassion. Wittmann et al. (2021) evaluated communication skills and found it particularly beneficial when fostering relationships with community partners. In related research, Cheng (2020) examined community relationships and found that officers in the study could show tendencies to resist the necessary change to effect substantive change.

Gong et al. (2020) quantitatively examined the role that adaptability plays in the career of law enforcement officers. They found adaptability to be especially impactful among officers with lower goal-self concordance. Similarly, Bennell et al. (2022) found that adaptability is a distinguishing factor in determining excellence among officers. In related research, Moreno et al. (2024) studied how resiliency training affected performance indicators and found a positive correlation. Similarly, Eliasson (2021) found that life experience positively affected policing.

Overall, the current literature clearly shows that the ideal officer cannot be defined solely by a single demographic or perspective. Preferred traits are a combination of self-identification, conscious decisions and behaviors, and perceptions from outside sources (McLean et al., 2020; Simon, 2023; Strah et al., 2023). This meandering definition opens an opportunity for researchers to examine the perceived ideal characteristics of law enforcement officers from various perspectives individually. That is to say, there is a research gap in which each perspective can be isolated and studied. In the current study, which is just one in a planned research series, the authors examined the perspectives, experiences, and opinions of the law enforcement officers’ peers. Fellow police officers provided the data for research examining the ideal characteristics of law enforcement officers.

## Problem statement

The problem this study seeks to address is identifying the perceived characteristics of the ideal law enforcement officer. Community members may define positive attributes in vastly different terms than law enforcement officers perceive those same traits. Even more compelling is how membership within the law enforcement community impacts the sought-after characteristics of their coworkers. The researchers in this study aim to find emerging themes regarding how law enforcement officers view the traits they would desire in their workplace. How a young officer perceives the ideal traits of a police officer may differ significantly from that of a police supervisor. Identifying and differentiating how separate groups define what good looks like among law enforcement officers is crucial.

## Research question

How do law enforcement officers describe the attributes, characteristics, and qualities that combine to form the archetype of the ideal law enforcer?

## Methods

Upon receipt of university IRB approval (#23–015), researchers in the current study solicited 22 participants from a single law enforcement agency on the United States West Coast. The Principal Investigator contacted the agency administrators, who reviewed and approved access to their employees but deferred the final approval to the labor union. Once the labor union representatives reviewed and approved access to their members, they sent out the solicitation materials that directed potential participants to a website to screen for qualifications, present the consent statement, and collect email addresses. Those email addresses were used to contact qualified participants and schedule the qualitative interviews. The criteria used for participant screening included the following qualifiers:

- Participants must be a current law enforcement officer
- Participants must belong to one of the two following categories:

o Hold the rank of officer
o Hold a supervisory rank

The researchers scheduled semi-structured interviews with the participants via Microsoft Teams video conference software, which lasted approximately 30–45 min each (Appendix A). Each interview was recorded using the Teams software; however, the participants were directed to leave their web cameras off during the interview to maintain confidentiality. A total of 22 participants were interviewed during the data collection process.

Merriam and Tisdell (2015) helped introduce and popularize the basic qualitative study, which uses qualitative methodologies and data collection but is not limited to the five commonly used frameworks described and so often cited by Creswell and Poth (2018). As the proliferation of the basic qualitative study emerged, several researchers published peer-reviewed studies demonstrating how data saturation was discovered (Dunn and Moore, 2020; Oakes, 2022; Weir and Panesar-Aguilar, 2022) (Table 1).

**Table 1:** Participants.

Pseudonym	Role	Gender	Age	Race	Education	Years of experience
Damian	Officer	Male	39	W	Some College	17
Devin	Officer	Male	55	W	Some College	22
Emily	Officer	Female	36	A	Bachelors	10
Emma	Officer	Female	34	H	Bachelors	11
Eric	Officer	Male	54	H	Some College	19
Jack	Officer	Male	50	A	Associates	25
Jim	Officer	Male	43	H	Masters	12
Naomi	Officer	Female	36	A	Some College	16
Roxanne	Officer	Female	35	H	Masters	5
Scott	Officer	Male	31	W	Masters	6
Susan	Officer	Female	38	H	Bachelors	14
Vanessa	Officer	Female	51	W	Some College	28
Andrew	Supervisor	Male	58	W	Some College	30
Charles	Supervisor	Male	57	W	Masters	36
Derrick	Supervisor	Male	43	W	Bachelors	18
Dylan	Supervisor	Male	53	W	Bachelors	27
Evelyn	Supervisor	Female	36	H	Masters	9
George	Supervisor	Male	52	H	Bachelors	29
Jerry	Supervisor	Male	45	W	Bachelors	24
Jill	Supervisor	Female	36	H	Masters	15
Nicole	Supervisor	Female	38	H	Bachelors	12
Valarie	Supervisor	Female	49	H	Some College	25

### Data analysis

Once the interviews were completed, the recordings were converted to transcriptions using Microsoft Teams transcription software. These transcripts were reviewed with the recordings to ensure accuracy. The researchers analyzed 732 pages of raw data using computer-aided qualitative data analysis software (NVivo 14) to conduct thematic analysis.

### Trustworthiness/rigor

Qualitative research requires trustworthiness to ensure the data is consistent and precise (Nowell et al., 2017). The primary component of trustworthiness is credibility (Lincoln and Guba, 1985). In the current study, credibility assurances came in the form of member checks. Participants were given the opportunity to review their transcripts to verify accuracy. Throughout the data analysis, the researchers maintained the necessary situational awareness through reflexivity and self-reflection (Karcher et al., 2024).

## Results

When this study’s participants described their ideal law enforcer, they did not limit the discussion to a singular role within policing. Given the various expectations of modern officers, the participants aligned their perspective of *ideal* with multiple roles. Some of the roles required emotional intelligence, while others relied on maturity and decision-making. The findings reflected the multiple roles with multiple definitions of ideal, depending on the volatility or sensitivity of a particular incident. When combining the list of traits and characteristics together, a clearer picture of the participants’ perceptions of the ideal law enforcer emerges.

The results of this analysis revealed there were consistencies in the way members of law enforcement officers described their perception of the ideal officer regardless of rank. The researchers found several themes that emerged from the thematic analysis of the data. Interestingly, there was little discussion of physical characteristics. Thematic analysis primarily revealed personality-related traits without discussing the physicality of police officers. The three themes that emerged were (1) Humanity and Emotional Intelligence, (2) Moral Courage, and (3) Resilience and Occupational Effectiveness. These three overarching themes contained several subthemes within each and will be discussed in the following section (Table 2).

**Table 2:** Primary themes and subthemes.

Primary theme	Sub-theme
Humanity and emotional intelligence	Compassion and Empathy
	De-escalation skills
	Effective communication
	Community relationships
Moral courage	Integrity
	Professionalism
	Humility
Resilience and occupational effectiveness	Adaptability
	Mental Strength
	Life experience

### Humanity and emotional intelligence

The theme of Humanity and Emotional Intelligence was discussed at length by the participants in this study. The collective definition achieved through commonalities among their interviews was the way in which officers initiated and maintained positive relationships with members of the community and fellow law enforcement employees. This was achieved through appropriate emotional and human connection. Four subthemes emerged from this perceived ideal characteristic of police officers. These four subthemes were Compassion and Empathy, De-escalation, Communication, and Community Relationships.

#### Compassion and empathy

The subtheme of Compassion and Empathy emerged as the participants described the attributes necessary to exhibit a human connection with members of the public in situations that range from crisis to routine. The participants spoke about the importance of caring as an officer and the need to emotionally engage with community members who are in need.

Damian (White, Male, 17 years of experience) addressed the concept of emergent situations when he said, “We see people on their worst days… we have to try to kind of break that emotional shock.” His statement shows how unimportant authority can be without emotional awareness.

Emily (Asian, Female, 10 years of experience) described her connection with people as a collaboration by saying, “You have to be in it with them, like, we are here together. It’s not just you, it’s not just me, it’s us.” This quote demonstrates a partnership with the area she polices. Using specific verbiage describing togetherness shows an understanding that an officer’s responsibilities include collaboration.

Scott (White, Male, 6 years of experience) presented an intriguing challenge in which he was required to transition from interviewing victims to arresting the offender, “Not that I’m saying I had to set aside any compassion, but I had to set aside the nice… from being exceptionally cordial to, now I have to go take that guy into custody.” His reflection highlights the struggle of officers transitioning between compassion and enforcement.

Andrew (White, Male, 30 years of experience) created a proverbial blank page with each interaction, saying, “I try to give everybody an opportunity, even if they are having their worst moment. We really do not need to make this worse than it already is.” His quote exemplifies the deliberate choice that officers can make to demonstrate compassion to diffuse volatile situations.

When viewed together, these participants describe how officers can see compassion and empathy as occupational requirements rather than mere personality traits. This subtheme supports the findings of Schuck and Rabe-Hemp (2024), who found that compassion was a highly desired trait for officers.

#### De-escalation

The next subtheme that emerged within Humanity and Emotional Intelligence was officers’ ability to de-escalate emotionally charged interactions. The participants discussed the perception that de-escalation should be a constant mindset rather than a mere policy to follow or a checkbox to tick. Susan (Hispanic, Female, 14 years of experience) recalled a de-escalation technique she was taught called verbal judo. “You use verbal judo and give them a voice and let them talk and ease their mind a little bit.” Her description of de-escalation as a strategy exemplifies a way to give community members a sense of control and ultimately adheres to a collaborative environment rather than an undesirable *us versus them* mentality. Andrew (White, Male, 30 years of experience) described the urgency associated with de-escalation by saying, “Find your way through the drama, the emotional conflicts that are happening. Sometimes, there’ll be multiple people there. Maybe they assaulted each other. You have got to figure it out real fast.” This quote personifies the speed at which policing decisions must be made and how prepared the officers must be to quickly quell a volatile encounter. Conversely, Eric’s (Hispanic, Male, 19 years of experience) perspective was more cautionary when he warned that de-escalation can be overemphasized and create unintended consequences. “The department is so hyped up about de-escalation, de-escalate, de-escalate, and that’s all they have in their head. De-escalate. De-escalate. De-escalate. That their de-escalation efforts are actually escalating situations that are turning into shootings.” This warning emphasizes a perception that rigid adherence to policy can be problematic when those decisions are not viewed through the lens of experience.

When these perceptions are combined, the importance of emotional intelligence in the form of sound judgment emerges. The officers paint a vivid picture of de-escalation as a primary focus that remains in the forefront during personal interactions instead of reluctant policy adherence.

#### Communication skills

Continuing with the subthemes that the participants discussed within the theme of Humanity and Emotional Intelligence, officers described the need for communication skills, but they also specified the nuance necessary to establish effective relationships. Through active listening, non-verbal communication, and adjusting messaging styles, participants demonstrated their desired traits of the ideal officer. Without effective skills to adequately communicate, officers feared an inability to defuse tense situations or connect with members of the community. Derrick (White, Male, 18 years of experience) emphasized that communication skills should be a priority when he said, “I think that one of the most important things in our job is being able to listen, a good listener and effective listener whether it’s through active listening or just using all your senses, you know you have to be able to pay attention to what’s going on around you.” This description shows how the unpredictability of policing can impact the importance of effective communication. Jill (Hispanic, Female, 15 years of experience) spoke of her experience with how the community desires to interact with law enforcement officers. “What I’ve found is most people just want to be heard.” Her statement addressed the emotional intelligence required to acknowledge and validate those who interact with law enforcers, which can demonstrate respect and foster legitimacy throughout an organization. Andrew (White, Male, 30 years of experience) described his method of maintaining communication during his radio calls: “There are different ways of communicating and different expectations, but your core foundation is still the same. You read the room, and you act accordingly.” By identifying the value of adjusting his tone and messaging, he can create a sense of equality and fairness in his interactions. Finally, Devin (White, Male, 22 years of experience) viewed the necessity of communication through the organizational lens. “When you stop talking to the community, they are left to search for information on their own.” This observation demonstrates how communication between law enforcers and the community is not merely an individual concern. Organizational reputations can be impacted by communication deficiencies.

Cumulatively, these lived experiences portray the significance of effective communication. The participants described it not only as an individual style but also as a professional tool that is necessary for promoting Peelian Principles like voluntary compliance and legitimacy (Kotsoglou and Oswald, 2025).

#### Community relationships

The remaining subtheme is Community Relationships. The interviews with participants provided a perspective that focused on traits that allowed officers to create a trusting and positive relationship with members of the public, regardless of the reason for contact. Emily (Asian, Female, 10 years of experience) demonstrated the way she prioritizes the importance of this trait by asking, “What’s the point of being a police officer if you do not have a community to serve? You cannot police nothing. You cannot police no people, so having those connections is important.” Her insight shows an understanding that the community is critical to success, and officers must fundamentally grasp the significance of external factors outside of the organization. Jack (Asian, Male, 25 years of experience) showed the value of fostering relationships that lead to positive results. “Even if they do not want to talk to you in person, you leave them a business card. We stopped all these crimes because they would call us or email us and let us know who was committing what crimes. He acknowledges that effective policing requires the assistance of those outside the agency in a collaborative relationship. Evelyn (Hispanic, Female, 9 years of experience) showed concern for the public’s perception of quality by saying, “They can at least trust that when we responded to their call for service, we actually cared, and we gave them the best service we could.” Naomi (Asian, Female, 16 years of experience) continued the topic of perception by demonstrating a desire to overcome media bias. “It is your job as that police officer to go to that radio call and help that community member realize that the media was wrong. I am here to help you.” These statements displays a realization that relationship-building is an ongoing process and must be consistent to pay dividends.

Much like the previous subthemes, the participants framed the relationship they foster with the community as a necessary component of being so-called *good*. Rather than a secondary option to enhance the existing professional abilities, building strong relationships was described as a primary goal.

### Moral courage

The second theme to emerge among the participants in their discussion of ideal characteristics in policing was the concept of Moral Courage. Participants bifurcated between physical courage and moral courage. Interestingly, physical courage as it relates to the ideal police officer was rarely mentioned. Instead, there were frequent discussions of moral courage as it pertains to honesty, ethical conduct, and professional behavior. The subthemes identified within Moral Courage were Integrity, Professionalism, and Humility.

#### Integrity

One of the most common themes to emerge in this study was Integrity. Of the 22 participants, 15 identified integrity as an ideal trait among law enforcement officers. Time after time, the participants’ interviews contained data supporting the idea that ethical standards are crucial to maintaining public trust. Emily (Asian, Female, 10 years of experience) spoke on the correlation between productivity and integrity held by the community when she said, “If they feel like they cannot trust us, they will not call us, and we will not be able to solve crimes or prevent crimes from happening.” Her quote appears to indicate that an ideal officer must rely on more than themselves for success. Eric (Hispanic, Male, 19 years of experience) differentiated between honesty and moral courage when he described how honest officers can be dissuaded from maintaining integrity with enough social pressure. “Someone can be very honest, but when you are in a position of pressure or peer pressure or so forth, some of the officers lack the moral courage to do it.” He identifies an intriguing aspect of integrity. External stressors can manipulate the decision-making process. Andrew (White, Male, 30 years of experience) focused on the concept that integrity encompasses more than doing the right thing; officers must also have the courage to stop wrong behavior. He said, “You just do not tolerate something you are not willing to deal with.” This statement continues along the subtheme, showing that not all integrity is internal. The ideal officer must also positively influence others. Jim (Hispanic, Male, 12 years of experience) emphasized how foundational integrity is within the occupation of policing. He said, “Integrity is the most important thing because you have to be honest with yourself. You have to be honest in everything you do.” Eric (Hispanic, Male, 19 years of experience) specified the importance of a morally courageous officer, describing them as “someone who does the right thing, no matter what, has the moral courage to stand up and make the hard decision, which is usually the right decision.” Both Jim and Eric highlight the universal nature of integrity and focus on the consistency that is necessary to embody that trait.

Previous subthemes were described as necessary components of so-called ideal officers, and integrity is no different, according to the participants. The difference lies in the ease with which the trait is displayed. Integrity, while necessary, was also portrayed as both a challenge and an active decision that can be difficult and lead to consequences. Regardless of the situation or rank, integrity appears to be a form of quiet leadership.

#### Professionalism

Participants identified Professionalism as a critical aspect of Moral Courage. They described the subtheme of Professionalism as an evident dedication to the job requirements and the high standards associated with maintaining those perceived ideal traits. Emily (Asian, Female, 10 years of experience) discussed professionalism in terms of physical appearance when she said the ideal officer is “adhering to the uniform standard as far as the hair goes. Their equipment is in decent condition, functional. Their duty belt is up against their body, and it’s not too tight or too loose.” She broached a rare description of ideal when she described physicality. Emma (Hispanic, Female, 11 years of experience) described the importance of leadership ability as a form of professionalism and moral courage. She emphasized that leadership and rank are not necessarily related when she said, “It does not matter if you wear the stripes or if you do not wear the stripes. I think regardless of being in any position or any role that you are in, you have to be a leader, and you have to be willing to speak up.” Her statement is an understanding of that the ideal law enforcer can be a leader, even if they are not a supervisor. Jill (Hispanic, Female, 15 years of experience) used customer service as a metaphor for professionalism. She said, “It absolutely is. It is customer service. We serve the community, and we have to make sure the community is happy with our service. Otherwise, what’s our purpose?” This indicates a perception that an officer’s effectiveness is judged by the public, not necessarily by the organization. Jack (Asian, Male, 25 years of experience) provided an example of a professional police officer when he spoke about the kind of officer that attracts the positive attention of other officers. “You always wanted to be the person that everybody wanted to work with.” He identifies a perspective that conflicts with Jill, in which the ideal officer should strive for internal validation.

The participants identified several characteristics from both internal and external sources that define professionalism. From physical appearance to the satisfaction of peers and the public, officers must display the traits that signal respect for their role as leaders in the community while still embracing their function as law enforcers.

#### Humility

The final subtheme to emerge under the theme of Moral Courage is Humility. Participants defined this trait as one who possesses the ability to learn from mistakes and can separate success from arrogance. Derrick (White, Male, 18 years of experience) described the law enforcement career as a learning process that requires humility from officers who are “open to criticism, that accept the criticism and learn from their mistakes, that understood that they are going to fail and that should not be afraid to fail.” His statement underscores the importance of failing forward in a dynamic career like policing. Damian (White, Male, 17 years of experience) discussed the concept of officers who possess enough humility to understand they are not infallible. He said, “You have to be humble to learn on this job because you are not gonna always get it right. You’re gonna make mistakes… you are not gonna have all the right answers. You’re gonna need help.” This quote identifies how accepting criticism is crucial yet difficult in the process of learning. Dylan (White, Male, 27 years of experience) provided some character traits to avoid when considering career survival. He said, “You cannot be an arrogant, pompous cop. [Saying] I’m telling you what to do, and you will do it… If you walk in there arrogant and pompous and without any kind of humility… [it could] cost you your career.” He describes the false value in bravado over true confidence in one’s ability.

The participants discussed humility as a quiet strength rather than a deficiency or self-deprecation. The ideal officer appears to be aware of their limitations while simultaneously soliciting and accepting critical feedback. Conversely, those who demonstrate a lack of humility were perceived to be a liability within the organization.

### Resilience and occupational effectiveness

The final theme to develop in the data was the concept of Resilience and Occupational Effectiveness. Participants described the significance of the ability to adapt to challenging environments and the mental toughness that develops with maturity and experience. The subthemes that emerged under this concept were Adaptability, Mental Strength, and Life Experience.

#### Adaptability

The first subtheme to emerge under the concept of Resilience and Occupational Effectiveness was the idea of Adaptability. The officers who participated in this study identified the necessity of flexibility and efficiency in high-stress environments. Jim (Hispanic, Male, 12 years of experience) described these traits as critical thinking and said, “I do not want [officers] to be trapped in a box… I want you to think about every perspective, and I want you to think of every solution to the problem because there’s no one way to skin a cat.” His perception points to the ideal officer having an ability to analyze fluid incidents safely but thoroughly. Scott (White, Male, 6 years of experience) alluded to the dynamic nature of decision-making in law enforcement as proverbial hats when he said, “You have to have a lot of hat options, and you have to know when and how to wear them.” Derrick (White, Male, 18 years of experience) also discussed the decision-making aspect of adaptability when he said, “Critically think about what’s happening in front of you that you can solve [the issue] to try to do it in a manner where it’s not gonna repeat itself and somebody else is gonna have to deal with this.” He identifies that each decision tree is going to follow a different path, and a good officer must be able to avoid complacency. Devin (White, Male, 22 years of experience) spoke on the topic of training as a method of reducing stress through exposure to situations. He said, “Training can help a person with exposure that may help mitigate spontaneous flight-fight response when exposed to a new event or stimulus.” This statement describes how crucial exposure can be in preparing for dynamic encounters.

Adaptability was discussed by the participants through the lens of preparedness. The ideal officer must adhere to the adage of expecting the unexpected. In an occupation that demands such flexibility, a one-size-fits-all approach will not foster success in volatile situations. This flexibility applies to decision-making and personal interactions.

#### Mental strength

The next subtheme analyzed in the data was the idea of Mental Strength. Officers defined this concept as the psychological well-being and mental toughness required to navigate a career in law enforcement. Andrew (White, Male, 30 years of experience) described the variety of negative situations that can lead to stress when he said, “You have to comprehensively understand what is happening whether it’s an emotional thing, a deadly thing, a scary thing, a yucky thing… You have to be able to understand and comprehend what is happening and immediately switch your gear.” His description of the variety of negative stressors that officers encounter underscores the importance of wide-ranging resilience preparation. Vanessa (White, Female, 28 years of experience) identified how physical injuries can take a toll on wellbeing. She said, “At 28 years [of law enforcement experience], I’ve got so many injuries… I even pick up a sock wrong at this point. It could be a really bad situation… do you really wanna put yourself out there with all this?” While not included in the typical description of ideal, her inclusion of physicality broached the exception wherein participants rarely discussed physicality over behavioral traits. Scott (White, Male, 6 years of experience) described the questions officers must ask themselves when making decisions. “You have to be brave… you have to be strong-willed in the sense that when somebody calls you and needs your help, are you brave enough to help them based on the situation that might arise from it?” In his discussion, he identified the preparation that Andrew described. Emily (Asian, Female, 10 years of experience) spoke on the topic of physical fitness and exercise as a way of mitigating mental stress when she said, “There are a lot of benefits to staying in shape, not just your physical self but your mental health is a lot better, your emotional well-being is a lot better” Her quote not only identifies the challenges related to mental strength but also exemplifies a solution in physical fitness.

When analyzed together, the participants discussed mental strength through the lens of resiliency after being exposed to various levels of occupational trauma. The ability to mitigate the strain while maintaining professional composure emerged at the forefront. The physical and emotional demands identified by the participants pointed to the ideal officer’s need to plan ahead to ensure psychological readiness.

#### Life experience

The final concept under the theme of Resilience and Occupational Effectiveness was Life Experience. Officers firmly held to the belief that the lifetime spent before becoming a police officer molded the decisions made while enforcing laws. Additionally, the time spent as a police officer while getting older and gaining life experience informed the ability to interact with a diverse community. This impact was particularly significant in complex and unpredictable situations. Roxanne (Hispanic, Female, 5 years of experience) described the ability of younger officers to relate to and advise older members of the community. She asked, “How is a 21-year-old gonna give advice to this 50-year-old couple … or this gangster that’s been already in and out of jail and is older? … How does your life experience apply to them?” This sentiment underscores the importance of finding common ground during personal interaction within the community, which can be difficult when a young officer has not developed similar experiences to reference. Susan (Hispanic, Female, 14 years of experience) described how she has evolved over the years as a police officer compared to when she began. She said, “Now that I’m 38 years old, I have children of my own … that all plays a role.” She describes the process in which life experience develops, whether through family growth, age, or exposure outside of the occupational setting. Eric (Hispanic, Male, 19 years of experience) spoke about how much authority is given to officers at a young age when he said, “Life experience, I think, experience on the job counts for something, decision-making, maturity… you are giving a 22-year-old a gun and the power to arrest.” He identifies the severity of the challenge facing officers who are given the responsibility of making life-and-death decisions at such a young age. Finally, Naomi (Asian, Female, 16 years of experience) provided herself as an example of using the entirety of life experience. She said, “I say you use everything that you have from your upbringing, your experiences out in the field because that’s ultimately what’s gonna be able to guide you and help others.” Her quote which relates to evolving throughout a lifecycle, points to an introspective lens when describing the ideal officer.

The participants identified life experience as a necessary component of the ideal officer. The maturity and growth associated with the years before beginning a career in law enforcement provide stability and an ability to relate to a diverse population. As described, maturity leads to wisdom and sound judgment in the dynamic or unpredictable incidents faced by officers.

## Discussion

The research question posed by the authors aimed to answer was to discover how law enforcement officers describe the attributes, characteristics, and qualities that combine to form the archetype of the ideal law enforcer. Ultimately, three themes emerged: Humanity and Emotional Intelligence, Moral Courage, and Resilience and Occupational Effectiveness. These themes may initially appear to represent a conflicting view of the ideal law enforcer, but when viewed through a multiple-role heuristic, they combine to represent the flexibility of the modern officer. Whether the officer is assuming the role of enforcer or mediator will determine the respective definition of ideal.

The current literature provided a binary perspective of options for enforcers. The choice between so-called warriors and guardians was clear and distinct (Clifton et al., 2021; Inzunza and Wikström, 2020; McLean et al., 2020; Murphy and McCarthy, 2024). The current study refuted the binary nature of policing by providing a combination of characteristics from each of the guardian or warrior columns; however, the findings supported characteristics from each option when viewed from the perspective of individual traits. Police officers, and humans in general, are not merely a sum of individual characteristics, and these traits can evolve (Haehner et al., 2024). They are a combination of attributes that form the whole person, and these attributes should not be viewed myopically.

### Humanity and emotional intelligence

The findings within this theme revealed a departure from the stereotypical toughness associated with American law enforcement officers, and the participants instead opted for more emotional capability. The participants placed significant value in the ability to transition between the highly charged tactical situations to the so-called softer side of policing, where communication, compassion, and empathy became the ideal characteristics. Four subthemes emerged. The first of these was Compassion. The participants identified compassion as a highly desired trait, which supported the findings of Schuck and Rabe-Hemp (2024), who found that compassion was a valued characteristic in policing. Additionally, Sunde’s (2024) previous finding that de-escalation was closely related to compassion was supported by the findings in the current study. Regarding the subtheme of Communication Skills, the current study found that to be a desired trait among law enforcement officers, which supported the findings of Wittmann et al. (2021). Conversely, the data in the current study refuted the findings of Cheng (2020), who found that officers can be hesitant to engage in substantive change related to relationships with the community. The participants in the current study identified community relationships as a crucial aspect of ideal policing.

### Moral courage

The findings within the theme provided insight into the significance officers place on the ability to prioritize integrity regardless of the professional, societal, or peer-based consequences. The importance of the courage required to set aside these risks and do the right thing emerged as participants described how crucial maintaining public trust is in relation to self-efficacy and mission success. The theme of Moral Courage, specifically the subtheme of humility, is most aptly applied to the research of Sjöberg et al. (2024). They found that a low level of vulnerability was ideal, whereas the current study found that willingness to admit mistakes and ignorance was a desired trait among fellow officers. When continuing to examine findings within the theme of Moral Courage, Integrity emerged as a subtheme. As the participants listed integrity as one of the ideal characteristics, it supported the findings of Menton et al. (2024) when they examined the lack of integrity among recruit officers as a correlate for field performance. Finally, the subtheme of professionalism emerged as a desired characteristic for law enforcement officers. This finding supports the research of Azzahrah et al. (2024), who found that officers with a high level of technical skill and knowledge are more respected than those who do not possess those attributes.

### Resilience and occupational effectiveness

The final theme that emerged from the data was Resilience and Occupational Effectiveness. Officers discussed the value placed on the ability to endure the physical and mental challenges experienced in repeated exposure to highly charged incidents, which can take a toll on an officer. The participants identified Adaptability within this concept as a beneficial aspect among law enforcers. These data support the findings of Bennell et al. (2022) and Gong et al. (2020). The participants discussed the topic of Mental Strength, and this emerged as a crucial aspect of the Resilience theme, which supported the findings of Moreno et al. (2024), who found that resiliency training has a positive correlation with performance indicators among police officers. The final finding within this theme is Life Experience. Officers heavily favored a law enforcer who has significant life experience before and after entering a policing career. Not surprisingly, this data supported the findings of Eliasson (2021), who also found that life experience prior to a career in law enforcement was a desired characteristic.

The participants were varied in the traits they perceived to be ideal in a law enforcer. There was a sense of normative conflict among their desired characteristics. On the one hand, the participants viewed quiet leadership, the courage to make difficult ethical decisions, and life experience as the attributes they viewed as ideal. These descriptions, while noble, can be viewed as internalized desires more than organizational demands. On the other hand, participants readily identified other traits that aligned with organizational values in the form of de-escalation, community relations, and problem-solving. The reflections of the officers underscore the conflict that can result from the need to fulfill multiple occupational roles and personal desires while still addressing the demands of their community and their agency.

### Limitations and recommendations for future research

While the current study adds to the body of knowledge, limitations are present. Generalizability is one limitation due to sampling. Participants self-selected into the study based on recruitment materials. Those who self-selected to participate could have different characteristics and ideals than their fellow officers. Additionally, the sample was comprised of law enforcement personnel from the West Coast of the United States. Due to the regional nature of the sample, the data collected may not indicate the ideology from other locations across the United States. Lastly, the findings from this study might not be representative of law enforcers in other countries.

As the researchers collected the data through interviews with current law enforcement officers, the span of experience and age became apparent. The desired characteristics of a younger officer with little experience may vastly differ from that of an officer with significant tenure. This is to say, the experiences of a 22-year-old officer may not be comparable to that of a 54-year-old officer planning for retirement after a 30-year career. These potential differences create an intriguing opportunity for future research. The career cycle of law enforcement officers should be researched through the lens of which characteristics are perceived to be ideal by new officers as compared to those of more experienced officers. Additionally, demographic-based analysis of the data set may identify distinctly different themes. Female officers, officers of color, or officers with higher levels of education may also provide intriguing descriptions of their perceived ideal law enforcer. Further, quantitative analysis of perceived ideal characteristics could lend additional insight into the qualitative findings presented in the current study.

## Conclusion

The researchers in the current study collected data through semi-structured interviews to help determine the ideal characteristics of law enforcers as perceived by officers themselves. These data presented three emerging themes: (1) Humanity and Emotional Intelligence, (2) Moral Courage, and (3) Resilience and Occupational Effectiveness. In examining the findings compared to the current literature, much of the data supported previous research findings. Intriguingly, the population in the study, police officers, rarely identified physicality among the desired qualities of the ideal law enforcer. Instead, they almost exclusively described personality-related traits without specific prompting from the researchers.

The researchers ultimately identified that the participants in this study maintain various perspectives of the ideal law enforcer rather than a singular archetype or the binary nature of guardian and warrior. Using multiple-role heuristic, the significance of understanding that transition and flexibility are crucial in modern policing. Not only do officers face different challenges with each type of public interaction, but these changes also potentially require a transition from one definition of ideal to a vastly different and conflicting definition of ideal. Understanding these complexities ultimately informs organizational policy and public scrutiny.

## Data Availability

The raw data supporting the conclusions of this article will be made available by the authors, without undue reservation.

## Appendix A: Semi-Structured Interview Guide

- Tell me about yourself, your age, current rank/position, and how long you have been in law enforcement.
- Think about law enforcement. Then think about the word successful. What do you see?
- What comes to mind when you picture the ideal law enforcement officer.
- Think about an incident where you saw someone do something that you thought was ideal in law enforcement and tell me about it.
- How can the “ideal” officer be developed? Are they born or made?
- Tell me about the benefits that are gained from being ideal in the field of law enforcement.
- Thinking about your current assignment, what traits are most important for an ideal law enforcement officer?
- What has changed about your idea of the ideal law enforcement officer since you first came on the job?
- How do you think newer officers view the ideal law enforcement officer compared to officers with more time on the job or supervisors?
- As a [specific rank], what has changed about your definition of the ideal law enforcement officer?
- When you think about the news and the internet, how have media and popular culture influenced the public’s idea of the ideal law enforcement officer? Give me some examples.
- What’s getting in the way of or stopping officers from being ideal?
- How is the ideal officer different in your area than other parts of the country where ideal may look different?

## References

[ref1] Azzahrah S. Tambun A. C. T. Balqis A. R. Prasna A. D. (2024). Ethics in law enforcement: analyzing the police professional code of ethics. J. Legal Cult. Anal. 3, 41–56. doi: 10.55927/jlca.v3i1.7263

[ref2] Bailey K. Lowder E. M. Grommon E. Rising S. Ray B. R. (2022). Evaluation of a police–mental health co-response team relative to traditional police response in Indianapolis. Psychiatr. Serv. 73, 366–373. doi: 10.1176/appi.ps.202000864, PMID: 34433289

[ref3] Bell S. Adams J. (2023). Do cops still care? A phenomenological exploration of narcotics (de) criminalization and officer discretion in Southern California. Am. J. Qual. Res. 7, 159–181. doi: 10.29333/ajqr/13730

[ref4] Bennell C. Jenkins B. Blaskovits B. Semple T. Khanizadeh A. J. Brown A. S.. (2022). Knowledge, skills, and abilities for managing potentially volatile police-public interactions: a narrative review. Front. Psychol. 13:818009. doi: 10.3389/fpsyg.2022.818009, PMID: 35330722 PMC8940200

[ref6] Charman S. Newiss G. Smith P. Inkpen R. Ilett C. Ghaemmaghami A.. (2022). ‘Giving the right service to different people’: revisiting police legitimacy in the Covid-19 era. Polic. Soc. 33, 348–365. doi: 10.1080/10439463.2022.2113785, PMID: 40101104

[ref7] Cheng T. (2020). Input without influence: the silence and scripts of police and community relations. Soc. Probl. 67, 171–189. doi: 10.1093/socpro/spz007, PMID: 31666754

[ref8] Chowdhury F. (2019). Application of rubrics in the classroom: a vital tool for improvement in assessment, feedback, and learning. Int. Educ. Stud. 12, 61–68. doi: 10.5539/ies.v12n1p61

[ref9] Clifton S. Torres J. Hawdon J. (2021). Examining guardian and warrior orientations across racial and ethnic lines. J. Police Crim. Psychol. 36, 436–449. doi: 10.1007/s11896-020-09427-6

[ref10] Cobbina-Dungy J. E. Jones-Brown D. (2023). Too much policing: why calls are made to defund the police. Punishment Soc. 25, 3–20. doi: 10.1177/14624745211045652

[ref11] Cook S. J. Fortunato D. (2023). The politics of police data: state legislative capacity and the transparency of state and substate agencies. Am. Polit. Sci. Rev. 117, 280–295. doi: 10.1017/S0003055422000624

[ref12] Creswell J. Poth C. (2018). Qualitative inquiry & research design: Choosing among five approaches. 4th Edn. Thousand Oaks, California: Sage Publications.

[ref13] Dunn A. L. Moore L. L. (2020). Significant learning of peer-mentors within a leadership living-learning community: a basic qualitative study. J. Leaders. Educ. 19, 64–75. doi: 10.12806/V19/I2/R5

[ref14] Eliasson M. (2021). Experience, seniority, and gut feeling—a qualitative examination of how Swedish police officers perceive they value, evaluate, and manage knowledge when making decisions. Front. Educ. 6:731320. doi: 10.3389/feduc.2021.731320, PMID: 40207237

[ref15] Fine A. D. Padilla K. E. Tom K. E. (2022). Police legitimacy: identifying developmental trends and whether youths’ perceptions can be changed. J. Exp. Criminol. 18, 67–87. doi: 10.1007/s11292-020-09438-7

[ref16] Gong Z. Yang J. Gilal F. G. Van Swol L. M. Yin K. (2020). Repairing police psychological safety: the role of career adaptability, feedback environment, and goal-self concordance based on the conservation of resources theory. SAGE Open 10, 1–11. doi: 10.1177/2158244020919510

[ref17] Haehner P. Bleidorn W. Hopwood C. J. (2024). Examining individual differences in personality trait changes after negative life events. Eur. J. Personal. 38, 209–224. doi: 10.1177/08902070231156840

[ref5] Hooper T. Woods K. Fallon K. Chilton H. Dennis R. Sedgwick A., (2024). Investigating primary and secondary school staff views on the level of need for educational psychology services in England:“What does good look like?”. Educ. Psychol. Pract. 40, 220–236. doi: 10.1080/02667363.2024.2319080

[ref18] Huff J. (2021). Understanding police decisions to arrest: the impact of situational, officer, and neighborhood characteristics on police discretion. J. Crim. Just. 75:101829. doi: 10.1016/j.jcrimjus.2021.101829

[ref19] Inzunza M. Wikström C. (2020). European police recruits’ views on ideal personal characteristics of a police officer. Polic. Soc. 30, 1243–1262. doi: 10.1080/10439463.2019.1685514

[ref37] Karcher K. McCuaig J. King-Hill S. (2024). (Self-) reflection/reflexivity in sensitive, qualitative research: A scoping review. Int. J. Qual. Methods. 23. doi: 10.1177/16094069241261860

[ref20] Kotsoglou K. N. Oswald M. (2025). Falling behind the PACE: lie detectors, policing and lack of foreseeability–an FOI-based study. Leg. Stud. 45, 58–78. doi: 10.1017/lst.2024.43, PMID: 40166671

[ref21] Krebs R. Rothstein B. Roelle J. (2022). Rubrics enhance accuracy and reduce cognitive load in self-assessment. Metacogn. Learn. 17, 627–650. doi: 10.1007/s11409-022-09302-1

[ref22] Landoni M. Huibers T. Murgia E. Pera M. S. (2022). Ethical implications for children’s use of search tools in an educational setting. Int. J. Child-Computer Int. 32:100386. doi: 10.1177/17470161241272872

[ref23] Lincoln Y. S. Guba E. G. (1985). Naturalistic inquiry, vol. 9: SAGE Publications, 438–439.

[ref24] Manzanero A. L. Quintana J. M. Contreras M. J. (2015). (the null) importance of police experience on intuitive credibility of people with intellectual disabilities. Res. Dev. Disabil. 36, 191–197. doi: 10.1016/j.ridd.2014.10.009 25462479

[ref9003] McCarthy M. McLean K. Alpert G. (2024). The influence of guardian and warrior police orientations on Australian officers’ use of force attitudes and tactical decision-making. Police Q. 27, 187–212. doi: 10.1177/10986111231189857

[ref25] McLean K. Wolfe S. E. Rojek J. Alpert G. P. Smith M. R. (2020). Police officers as warriors or guardians: empirical reality or intriguing rhetoric? Justice Q. 37, 1096–1118. doi: 10.1080/07418825.2018.1533031

[ref26] Menton W. H. Corey D. M. Ben-Porath Y. S. (2024). Correlates of the selection validation survey with police officer field performance. J. Pers. Assess. 106, 37–48. doi: 10.1080/00223891.2023.2182692, PMID: 36857474

[ref27] Merriam S. B. Tisdell E. J. (2015). Qualitative research: A guide to design and implementation. San Francisco, California: John Wiley & Sons.

[ref28] Moreno A. F. Karanika-Murray M. Batista P. (2024). Resilience training programs with police forces: a systematic review. J. Police Crim. Psych. 39, 227–252. doi: 10.1007/s11896-023-09633-y

[ref29] Murphy K. McCarthy M. (2024). Guardian versus warrior cops: predicting officers’ support for procedural justice and coercive policing. Polic. Soc. 34, 1011–1030. doi: 10.1080/10439463.2024.2357653

[ref30] Nowell L. S. Norris J. M. White D. E. Moules N. J. (2017). Thematic analysis: striving to meet the trustworthiness criteria. Int J Qual Methods 16, 1–13. doi: 10.1177/1609406917733847, PMID: 40182056

[ref31] Oakes T. (2022). Instructional designers’ strategies for online activities enhancing self-directed learning: a basic qualitative study. Perform. Improv. 61, 39–50. doi: 10.56811/PFI-22-0002

[ref32] Oliveira T. R. Jackson J. (2021). Legitimacy, trust, and legal cynicism a review of concepts. Tempo Social 33, 113–145. doi: 10.11606/0103-2070.ts.2021.191381

[ref33] Perry G. Jonathan-Zamir T. (2020). Expectations, effectiveness, trust, and cooperation: public attitudes towards the Israel police during the COVID-19 pandemic. Policing 14, 1073–1091. doi: 10.1007/s11292-024-09622-z PMC7665771 40504219

[ref34] Plummer J. A. Morales M. G. Dettlaff A. J. (2024). Defund, abolish, or reform: MSW student perceptions of police reform. J. Policy Pract. Res. 5, 194–208. doi: 10.1007/s42972-024-00108-w, PMID: 40207182

[ref36] Schuck A. M. Rabe-Hemp C. E. (2024). Women police, legitimacy, and ethics of care. Policing 18, 1–11. doi: 10.1093/police/paae006

[ref38] Simon S. J. (2023). Training for war: academy socialization and warrior policing. Soc. Probl. 70, 1021–1043. doi: 10.1093/socpro/spab057

[ref39] Sjöberg A. Larsson A. C. Tedeholm P. G. (2024). Using the five-factor model of personality to identify an optimal SWAT team member. Int. J. Police Sci. Manag. 27, 3–15. doi: 10.1177/14613557241245623, PMID: 40182056

[ref40] Strah B. M. Pollock J. M. Becker L. T. (2023). Shifting from warriors to guardians: officer reflections on law enforcement training in Washington state. Crime Delinq. 69, 439–463. doi: 10.1177/00111287221117488

[ref41] Sunde H. M. (2024). How does it end well? An interview study of police officers’ perceptions of de-escalation. Nordic J. Stud. Polic. 11, 1–21. doi: 10.18261/njsp.11.1.1

[ref42] Ten Eyck M. F. (2024). The “police personality”: is it real? Police Q. 27, 271–291. doi: 10.1177/10986111231193032, PMID: 40182056

[ref43] Townsend K. Loudoun R. (2024). Line managers and extreme work: a case study of human resource management in the police service. Int. J. Hum. Resour. Manag. 35, 1763–1785. doi: 10.1080/09585192.2023.2251875, PMID: 40101104

[ref44] Weir A. Panesar-Aguilar S. (2022). Using Alexa to differentiate instruction in the special education classroom. Int. J. Multidisciplin. Res. Analysis 5, 472–477. doi: 10.47191/ijmra/v5-i2-34

[ref45] Wittmann L. Jörns-Presentati A. Groen G. (2021). How do police officers experience interactions with people with mental illness? J. Police Crim. Psychol. 36, 220–226. doi: 10.1007/s11896-020-09398-8

[ref46] Zakimi N. Greer A. Butler A. (2022). Too many hats? The role of police officers in drug enforcement and the community. Policing 16, 615–629. doi: 10.1093/police/paab082

